# Understanding the main barriers to immunization in Colombia to better tailor communication strategies

**DOI:** 10.1186/1471-2458-14-669

**Published:** 2014-06-30

**Authors:** Diego Alejandro García L, Martha Velandia-González, Silas Pierson Trumbo, M Cristina Pedreira, Pamela Bravo-Alcántara, M Carolina Danovaro-Holliday

**Affiliations:** 1 Expanded Program on Immunization, Carrera 13 No. 32-76, Bogotá, Colombia; 2 Comprehensive Family Immunization Unit, Pan American Health Organization, 525 23rd St., NW Washington, DC 20037, USA

**Keywords:** Immunization programs, Colombia, Immunization services, Barriers to immunization, Communication strategies

## Abstract

**Background:**

The Expanded Program on Immunization (EPI) in Colombia has made great advances since its inception in 1979; however, by 2010 vaccination coverage rates had been declining**.** In 2010, the EPI commissioned a nationwide study on practices on immunization, attitudes and knowledge, perceived service quality, and barriers to childhood immunization in order to tailor EPI communication strategies.

**Methods:**

Colombia’s 32 geographical departments were divided into 10 regions. Interviewers from an independent polling company administered a survey to 4802 parents and guardians of children aged <5 years in these regions. To better assess barriers to vaccination, the study was designed to have 70% of participants who had children with incomplete vaccination schedules. Explanatory factorial, principal component, and cluster analyses were performed to place participants into a group (segment) representing the primary category of reasons respondents offered for not vaccinating their children. Types of barriers were then compared to other variables, such as service quality, communication preferences, and parental attitudes on vaccination.

**Results:**

Although all respondents indicated that vaccines have health benefits, and 4738 (98.7%) possessed vaccination cards for their children, attitudes and knowledge were not always favorable to immunization. Six groups of immunization barriers were identified: 1) factors related to caregivers (24.4%), 2) vaccinators (19.7%), 3) health centers (18.0%), 4) the health system (13.4%), 5) concerns about adverse events (13.1%), and 6) cultural and religious beliefs (11.4%); groups 1, 5 and 6 together represented almost half (48.9%) of users, indicating problems related to the demand for vaccines as the primary barriers to immunization. Differences in demographics, communication preferences, and reported service quality were found among participants in the six groups and among participants in the 10 regions. Additionally, differences between how participants reported receiving information on vaccination and how they believed such information should be communicated were observed.

**Conclusions:**

Better understanding immunization barriers and the users of the EPI can help tailor communication strategies to increase demand for immunization services. Results of the study have been used by Colombia’s EPI to inform the design of new communication strategies.

## Background

The Expanded Program on Immunization (EPI) in Colombia operates under the Ministry of Health and Social Protection^a^ (MSPS in Spanish) and within the General Health and Social Security System (SGSSS in Spanish). The country’s immunization schedule includes nine vaccines: Bacille Calmette-Guerin (BCG); diphtheria, pertussis, and tetanus (DPT) vaccine; Hepatitis B; influenza; measles, mumps, and rubella (MMR) vaccine; oral poliovirus vaccine (OPV); pentavalent (DPT-*Haemophilus influenzae* type b or Hib); rotavirus; and yellow fever. These vaccines are provided free of charge to all citizens, and offer protection against 13 pathogens: Diphtheria, Hepatitis B, Hib, influenza, measles, mumps, pertussis, poliovirus, rotavirus, rubella, tetanus, tuberculosis, and yellow fever. According to a recent external evaluation of the program, the EPI has more than tripled its budget for vaccine purchases from $32.6 million in 2008 to $143 million in 2012 [1]. To reach unvaccinated and undervaccinated children, the EPI has conducted vaccination days and campaigns, established fixed vaccination posts, and sent vaccination brigades to hard-to-reach areas.

Vaccination coverage rates in Colombia have decreased over the last decade. From 2003–2009, administrative coverage^b^ of BCG, third dose of oral polio vaccine (OPV3), and third dose of diphtheria, pertussis, and tetanus vaccine (DPT3) ranged from 89-97% [2]. By 2011, BCG and OPV3 coverage rates had fallen to 80.9% and 84.3%, respectively, with lower levels reported in many areas [3]. Previous studies have linked undervaccination in Colombia to poor service quality [4-7], lack of parental knowledge of the vaccination schedule [4,7], lack of a regular physician [4], low insurance coverage [5,6], inadequate health worker knowledge [6], and ineffective communication strategies [7]. In addition, inaccurate population estimates (i.e. issues related to the denominator) may have affected coverage trends [1].

A successful communication intervention on immunization can be defined as an informed, purposeful, and repeatable strategy that motivates caregivers to seek or continue seeking immunization services for their children [8]. Strategies may include radio and television spots, educational workshops and sessions, printed materials (flyers, banners, posters), and clinical interactions between the parent and healthcare professional [8]. Common to all strategies is the need for careful research and planning [9]. Target populations, means of communication, and the content and frequency of messages must be determined and weighed against budgetary constraints. In this context, differences among users by age group, class, and geographic region must be taken into account. Additionally, user knowledge, attitudes, and past experiences associated with barriers to immunization should be considered in developing messages to overcome these barriers [9,10].

To better understand immunization barriers in Colombia and inform the design of tailored communication strategies aimed at improving immunization coverage levels, the EPI hired an independent polling company to conduct a study on factors inhibiting immunization among parents and guardians of children aged <5 years. Information was collected on user demographics, attitudes, knowledge, and practices; their perceptions about the quality of immunization services; the impact of existing communication strategies; and barriers to immunization. Based on this information, groups of reasons explaining why caregivers do not vaccinate their children were identified. Following this study, the EPI developed a plan to improve national communication strategies on immunization based on the barriers identified as related to parents/users.

## Methods

### Sampling

Participants were required to be adults responsible for the vaccination of at least one child aged <5 years. Because the study focused on the reasons parents could not or choose not to vaccinate their children, the study was designed to have approximately 70% of participants with children who had incomplete vaccination schedules, as corroborated by the interviewers who had been trained to assess if the child was up to date with immunization. Children with incomplete schedules were defined as those lacking at least one vaccine recommended in the national schedule for their age. If a participant had multiple children aged <5 years, interviewers collected information on all children in order to determine if reasons for undervaccination varied among siblings in the same household. Participants were excluded from the study if they failed to meet the criteria above or if they worked for a polling or public relations company.

**Figure 1:**
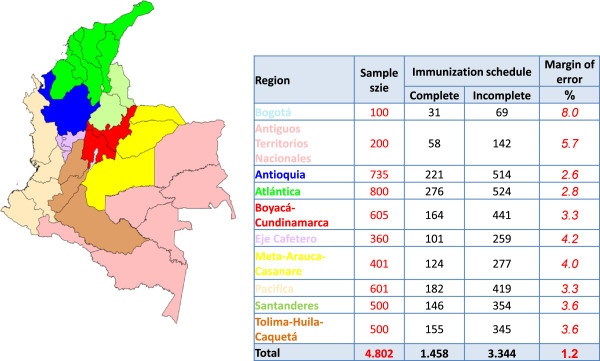
10 regions of Colombia with sample sizes and margins of error, May 2010.

Colombia’s 32 geographical departments were divided into 10 regions: Antiguos Territorios Nacionales, Antioquia, Atlántico, Bogotá, Boyacá-Cundinamarca, Eje Cafetero, Meta-Arauca-Casanare, Pacífica, Santanderes, and Tolima-Huila-Caquetá (Figure 1 and Additional file 1). The polling company employed a multi-stage sampling design to select participants. The MSPS provided a list of municipalities with administrative coverage <75% in each region as well as municipal population and vaccination coverage estimates. From this list, 3–10 municipalities in each region were chosen with probability proportional to the population of each municipality and inversely proportional to the coverage reported in that area. A total of 99 municipalities were selected. In each municipality, interviewers chose neighborhoods and blocks at random. Interviewers visited each home on the block to reach a quota of homes of children aged <5 years with complete (30%) and incomplete (70%) vaccination schedules. If the quota could not be completed from the block initially selected, interviewers moved to the next contiguous block. The overall sample had a margin of error of 1.2%, while the median margin of error for an individual region was 3.6%.

### Implementation

The surveying team consisted of one national director, several regional directors, approximately 200 interviewers and local-level supervisors, and an analytical team composed of statisticians, psychologists, and other professionals. To help design the study, the company conducted an initial qualitative evaluation, through focus groups, of the reasons that Colombians do not or cannot vaccinate their children.

Interviewers had extensive experience conducting opinion surveys, and the company had previously implemented health surveys. Training sessions were held to educate team members on technical topics related to vaccination and to show interviewers and supervisors how to read vaccination cards. During the sessions, team members reviewed the surveying tools to correct potential areas of misunderstanding. A pilot project was also held to help interviewers gain familiarity with the surveying tools.

Interviewers administered the questionnaire to participants in their homes from 18 May to 9 June 2010. Surveys typically lasted 25–35 minutes and were completed on weekday afternoons and throughout the day on weekends. Participants were guaranteed anonymity and provided informed verbal consent. Participants could discontinue interviews at any time and received no compensation for their time.

To ensure data quality, supervisors directly observed 40% of the interviews and reviewed all questionnaires. Questionnaires were then sent to regional field offices and to a national processing center in Bogotá for additional review. If a problem was detected at any point in the process (e.g., incomplete responses), the interviewer returned to the participant’s home to correct the issue. Validated data was coded and entered into a Microsoft Access database.

The independent polling company delivered the final report of the study to the Ministry of Health five months after the study had been officially commissioned.

### Questionnaire

To better understand the target population, define the variables to be measured in the study, design the questionnaire, and propose hypotheses on immunization barriers for testing in the study’s quantitative phase, the polling company conducted a pre-study evaluation of the intended target population. A total of 186 caregivers of incompletely vaccinated children aged <5 years living in municipalities with <75% coverage in the selected 10 regions were convened in focus groups of 10–12 persons. Moderators in each group led a conversation on parental, structural, cultural, and service-related factors that might inhibit immunization (e.g., unfriendly or discriminatory service). Discussions lasted approximately two hours. Unlike participants in the qualitative study, members of the focus groups were compensated for their time.

Based on input from EPI officials and the information gathered from the focus groups, the polling company developed a questionnaire for evaluating the causes of undervaccination in the country. The EPI reviewed the questionnaire to guarantee technical accuracy. The final survey contained 76 questions related to demographics; parental knowledge, attitudes, and practices on vaccination; user satisfaction; service quality; communication preferences and practices; and barriers to immunization. In Colombia, parents are required by law to present their children’s complete immunization histories for school enrollment [11]. Children with incomplete schedules are typically sent to health centers for vaccination; no child is denied the right to an education due an incomplete schedule. To measure compliance with the law, participants were asked whether they had been required to provide their children’s immunization histories to kindergartens or preschools. Participants were also asked two questions on appointments for vaccination: 1) whether an appointment for vaccination was required (an appointment should not be needed) and 2) whether health workers wrote the date that the child is next scheduled to be immunized on the vaccination card (health workers should note the date per EPI guidelines).

Various question types were used, including recall, dichotomous, open-ended, and multiple response questions [12]. Barriers to immunization were assessed with level-of-measurement questions in which participants ranked various options, and with level-of-agreement questions in which participants “agreed”, “disagreed”, or “neither agreed nor disagreed” with a statement concerning vaccination. An immunization barrier was defined as any factor that a caregiver considers to have prevented or may in the future prevent a child from being vaccinated.

### Data analysis

To measure factors inhibiting immunization, the polling company performed an exploratory factorial analysis, which is a multivariate technique of interdependence to explain the correlations between observed variables losing the least amount of information possible. The principal component analysis was used to determine causes for the variability of a set of continuous variables and rank them by importance. Based on a literature review and the results of the qualitative component done using focus groups, 30 potential vaccination barrier variables were included in the factorial analysis. Variables that had eigenvalues greater than one and could explain at least 60% of the variability were retained for the principal component analysis A cluster analysis was used to include each individual in a group, such that individuals of the same segment were similar to each other and different from others, especially on issues related to the reasons for non-vaccination, but keeping in mind that some demographic variables or topics covered in the questionnaire could present similarities between segments.

Sampling weights were then used to correct for oversampling in some regions, thereby ensuring that the number of participants in each segment reflected the distribution of the population in Colombia.

Lastly, the types of barriers were compared with variables on demographics, service quality, communication preferences, and parental attitudes on vaccination. A percentage for the overall sample was used as a reference point to identify each group’s salient features (e.g., 33.5% of participants in group one live in Atlántico versus 23.1% in the overall sample). Significant deviations from overall rates are highlighted in the results section of the paper. Other statistical analyses were not conducted due to the descriptive nature of the study. All data were processed and analyzed with version 13 of the Statistical Package for the Social Sciences Software (SPSS).

### Ethics

In Colombia, studies that do not seek to generate generalizable knowledge, but rather to answer specific questions of public health importance are considered “public health operational investigations.” As such, they are not subject to the IRB review process. However, and in order to share these results in a peer reviewed publication, the Ethics Committee of the National Institute of Health of Colombia. reviewed all the documentation related to this manuscript and the study methodology in a meeting held on July 11, 2013. The CERN determined that the study was exempt from an ethical review.

## Results

Approximately 50,000 individuals were contacted for participation in the study. Of these, approximately 5050 met the inclusion criteria and 4802 (95.0%) chose to participate. By geographic region, the median study population was 500 participants. Most participants were female (96.8%), aged 19–30 years (57.3%), subsidized users of the Colombian healthcare system (74.2%), and possessed at least a primary level of education (81.6%) (Table 1). Twenty-nine percent self-identified as Afro-Colombian and 8.6% as indigenous. Participants had a total of 5709 children aged <5 years.

**Table 1:** Characteristics of interviewed persons: Colombia, May 2010

**Characteristic**	**Participants (n = 4802) No. (%)**
***Sex of participants***	
Male	154 (3.2)
Female	4648 (96.8)
***Age (years)***	
14-18	355 (7.4)
19-25	1558 (32.4)
26-30	1198 (24.9)
31-40	1179 (24.6)
>40	512 (10.7)
***Relation to child***	
Mother	4217 (87.8)
Father	149 (3.1)
Grandparent	328 (6.8)
Other family member	95 (2.0)
Neighbor	9 (0.2)
No response/I don’t know	4 (0.1)
***Education***	
Less than primary	883 (18.4)
Primary only	843 (17.5)
Secondary incomplete	1194 (24.9)
Secondary complete	1429 (29.7)
Post-secondary	450 (9.4)
No response/I don’t know	3 (0.1)
***Marital Status***	
Single	1022 (21.3)
Married	966 (20.1)
Civil union	2489 (51.8)
Separated/divorced	240 (5.0)
Widowed	78 (1.6)
No response/I don’t know	7 (0.2)
Affiliation with health system*	
Subsidized	3561 (74.2)
Contributor	1015 (21.1)
Non-affiliated poor	127 (2.6)
Other	92 (1.9)
No response/I don’t know	7 (0.2)
***Ethnicity***	
Indigenous	412 (8.6)
Afro-Colombian	1393 (29.0)
Other	2957 (61.6)
No response/I don’t know	40 (0.8)
***Children***	
1	3151 (65.6)
2	1066 (22.2)
3	391 (8.2)
>4	194 (4.0)
***Age of child in study (n = 5709)***	
0-1 years	1179 (20.7)
1-2 years	1208 (21.1)
2-3 years	1049 (18.4)
3-4 years	1114 (19.5)
4-5 years	1159 (20.3)

*By law, all Colombians are entitled to health and immunization services. Following the reform of the country’s health system, citizens are considered contributivos (those able to pay for services) or subsidiados (those whose receive subsidized care). A small percentage of the population remains unaffiliated with the system.

### Practices

Of 4802 participants, 4738 (98.7%) possessed vaccination cards for their children. As required by the methodology, 3344 (69.6%) had at least one child with an incomplete vaccination schedule and 1610 (48.1%) were unaware that their child was undervaccinated (Table 2).

**Table 2:** **Practices, knowledge, and attitudes on vaccination and quality of immunization services, by region: Colombia, May 2010**★

**Indicators (agreement with given statement)**	**Total (n = 4802) No. (%)**	**Region**									
		**Bogotá (n = 100) No. (%)**	**Antiguos Territorios Nacionales (n = 200) No. (%)**	**Antioquia (n = 735) No. (%)**	**Atlántico (n = 800) No. (%)**	**Boyacá-Cundinamarca (n = 605) No. (%)**	**Eje Cafetero (n = 360) No. (%)**	**Meta-Arauca-Casanare (n = 401) No. (%)**	**Pacífica (n = 601) No. (%)**	**Santanderes (n = 500) No. (%)**	**Tolima-Huila-Caquetá (n = 500) No. (%)**
***Knowledge and attitudes***											
I know what disease each vaccine prevents	3077 (64.1)	87 (87.0)	**103 (51.5)**	464 (63.1)	679 (84.9)	363 (60.0)	249 (69.2)	256 (63.8)	358 (59.6)	296 (59.2)	**222 (44.4)**
My parents did NOT become sick and they were never vaccinated?	819 (17.1)	24 (24.0)	**75 (37.5)**	83 (11.3)	142 (17.8)	83 (13.7)	77 (21.4)	34 (8.5)	**190 (31.6)**	54 (10.8)	57 (11.4)
People living in rural areas do not need vaccines◆	596 (12.4)	15 (15.0)	**53 (26.5)**	65 (8.8)	90 (11.3)	69 (11.4)	49 (13.6)	29 (7.2)	**145 (24.1)**	53 (10.6)	28 (5.6)
***Practices***											
I have vaccination cards for all my children**	4738 (98.7)	99 (99.0)	197 (98.5)	726 (98.8)	794 (99.3)	601 (99.3)	360 (100.0)	400 (99.8)	586 (97.5)	493 (98.6)	482 (96.4)
I was NOT aware that my child was lacking one or more vaccines (n = 3344)✚	1610 (48.1)	**43 (62.3)**	**89 (62.7)**	111 (21.6)	232 (44.3)	251 (56.9)	128 (49.4)	**184 (66.4)**	**275 (65.6)**	175 (49.4)	122 (35.4)
I need an appointment to vaccinate my child◆	2178 (45.4)	19 (19.0)	104 (52.0)	**431 (58.6)**	241 (30.1)	235 (29.4)	**231 (64.2)**	143 (35.7)	290 (48.3)	248 (49.6)	236 (47.2)
I have once had to pay for a vaccine	150 (3.1)	3 (3.0)	3 (1.5)	**69 (9.4)**	10 (1.3)	18 (3.0)	2 (0.6)	8 (2.0)	13 (2.2)	14 (2.8)	10 (2.0)
I have forgotten at least one vaccination appointment	1133 (23.6)	**31 (31.0)**	**111 (55.5)**	102 (13.9)	232 (29.0)	182 (30.1)	47 (13.1)	39 (9.7)	**195 (32.4)**	100 (20.0)	94 (18.8)
***Service Quality***											
During my last visit to a health center, immunization services were “excellent” or “good”	4137 (86.2)	94 (94.0)	169 (84.5)	710 (96.6)	633 (79.1)	558 (92.2)	327 (90.8)	369 (92.0)	**437 (72.7)**	421 (84.2)	419 (83.8)
Healthcare workers tell me my child’s next vaccination appointment°°	4415 (91.9)	90 (90.0)	187 (93.5)	715 (97.3)	725 (90.6)	548 (90.6)	354 (98.4)	378 (94.3)	531 (88.3)	**428 (85.6)**	459 (91.8)
Healthcare workers tell me which vaccines/boosters my child has received°°	3733 (77.7)	88 (88.0)	**138 (69.0)**	635 (86.4)	655 (81.9)	511 (84.5)	292 (81.1)	304 (75.8)	471 (78.4)	**338 (67.6)**	**301 (60.2)**
Healthcare workers inform me of the risk of adverse events°°	4283 (89.2)	92 (92.0)	**160 (80.0)**	697 (94.8)	712 (89.0)	564 (93.2)	343 (95.3)	358 (89.3)	517 (86.0)	**372 (74.4)**	468 (93.6)
During my child’s last check-up, a physician reviewed my child’s vaccination card	3807 (79.3)	86 (86.0)	152 (76.0)	677 (92.1)	686 (85.8)	514 (85.0)	317 (88.1)	302 (75.3)	**369 (61.4)**	**307 (61.4)**	397 (79.4)
I had to present my child’s vaccination card as a prerequisite for her entry into preschool or kindergarten	2930 (61.0)	61 (61.0)	**89 (44.5)**	409 (55.6)	630 (78.8)	379 (62.6)	240 (66.7)	**171 (42.6)**	330 (54.9)	364 (72.8)	**257 (51.4)**

★Values in this table reflect participant responses in each of the 10 regions evaluated. Bold values reflect deviations from rate in overall study population (difference of >8 points).

**Participants were required to provide interviewers with a copy of the child’s vaccination card.

✚Total include only those participants with at least one child with an incomplete immunization schedule (n = 3344). Percentages based on regional totals of children with incomplete schedules: Bogotá (69), Antiguos Territorios Nacionales (142), Antioquia (514), Atlántica (524), Boyacá-Cundinamarca (441), Eje Cafetero (259), Meta-Arauca-Casanare (277), Pacífica (419), Santanderes (354), and Tolima-Huila-Caquetá (345).

◆For the purposes of this table, totals include respondents who indicated they agreed with statement given. Participants who “neither agreed nor disagreed,” “disagreed,” or chose not to respond were excluded.

°°For the purposes of this table, totals include respondents who indicated that event occurred “always” or “almost always.” Participants who indicated event occurred “sometimes,” “rarely,” or “never” were excluded.

Overall, 1133 (23.6%) participants reported having forgotten at least one vaccination appointment. Participants reported being most likely to forget appointments in Antiguos Territorios Nacionales (55.5%) and Pacífica (32.4%) and least likely to do so in Eje Cafetero (13.1%) and Meta-Arauca-Casanare (9.8%). Nationwide, 2178 (45.4%) caregivers indicated that they needed an appointment to vaccinate their child. The regions where most participants said they needed an appointment were Antiguos Territorios Nacionales (52.0%), Antioquia (58.6%), and Efe Cafetero (64.2%).

A small percentage of participants (n = 150, 3.1%) indicated that they once had to pay for vaccines. This finding was consistent among regions, except in Antioquia where 69 (9.4%) caregivers reported once paying for vaccination. In most cases (n = 82/150, 54.7%), participants said they had to pay for pneumococcal conjugate vaccine (PCV), which was not included in the EPI schedule at the time of the study.

### Attitudes and knowledge

All 4802 respondents indicated that vaccines have health benefits. However, 819 (17.1%) said that “their parents were never vaccinated and never became sick” and 596 (12.4%) said “that people living in rural areas do not need vaccines.” Among vaccines in the national schedule, participants recognized the measles-mumps-rubella (MMR) and polio vaccines most frequently (57.0% and 53.4%, respectively) and rotavirus vaccine least frequently (19.0%). Residents of Atlántico and Bogotá reported greater knowledge of vaccines. Residents of Antiguos Territorios Nacionales and Tolima-Huila-Caquetá reported less knowledge. Inconsistencies were found between participants’ self-described and actual knowledge of vaccines: 3077 (64.1%) claimed to know which disease each vaccine prevented, but the average respondent named only four diseases. Of the 1725 participants claiming to lack this knowledge, 1056 (61.2%) said that health workers failed to provide them with clear information.

### Service quality

A total of 4137 (86.2%) participants considered their last visit to a vaccination center “good” or “excellent”, 567 (11.8%) considered it “average”, and 94 (2.0%) considered it”bad” or “very bad.” Nationwide, 4415 (91.9%) participants reported receiving a “next vaccination” appointment, 3733 (77.7%) reported being told which vaccine their child had received, and 4283 (89.2%) reported being informed of the possibility of adverse reactions.

Service quality varied by region, with poorest service reported in Antiguos Territorios Nacionales, Pacífica, Santanderes, and Tolima-Huila-Caquetá. When participants were asked whether they had to present proof of their child’s immunization status for school enrollment, 2930 (61.0%) said “yes.” Participants reported that a greater proportion of nurses (n = 4438, 92.4%) than physicians (n = 3807, 79.3%) reviewed their child’s vaccination card during the last wellness visit, and 3442 (71.7%) participants reported that they had been asked for the card when visiting a health facility for a purpose other than vaccination. Lastly, 2228 (46.4%) indicated they had recently faced circumstances making vaccination difficult or impossible. Such circumstances included distance from health centers (n = 809), lack of vaccines (n = 591), limited days/hours of operation (n = 423), and refusal of healthcare personnel to open a vaccine vial for one child (n = 390).

### Communication strategies

**Figure 2:**
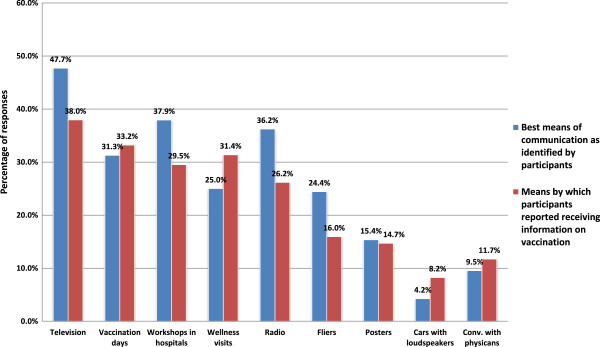
Means of communication, practices versus best means as identified by participants: Colombia, May 2010* *. Participants were able to identify multiple ways that they had received information on immunization as well as multiple ways that they believed were the best means for receiving such information.

Participants believed that hospitals (54.1%), the municipal government (34.2%), and the departmental government (33.4%) were responsible for publicizing immunization. The three most common ways parents reported receiving information on immunization were television (38.0%), vaccination days/campaigns (33.2%), and conversations with health workers during pediatric wellness visits (31.4%). Television was reported as the primary means of communication in three regions (Atlántico, Bogotá, Boyacá-Cundinamarca), pediatric wellness visits in three (Antioquia, Pacífica, Santanderes), vaccination days/campaigns in two (Eje Cafetero, Meta-Arauca-Casanare), radio in one (Antiguos Territorios Nacionales), and mobile loudspeakers on cars in one (Tolima-Huila-Caquetá). In comparing these responses to answers on the best ways to publicize immunization, differences were observed at the regional and national levels. Figure 2 shows that 1740 (36.2%) participants considered radio a preferable means of communication, while only 1257 (26.2%) indicated they had received information by radio. Similarly, 396 (8.2%) reported learning of immunization through mobile loudspeakers, but only 204 (4.2%) considered loudspeakers a valuable means of communication. Overall, only four respondents chose the Internet as their preferred means of receiving information on vaccination.

Participants were asked to evaluate the efficacy of current communication efforts. A total of 3335 (69.5%) participants agreed with the statement, “Messages exist that motivate parents to vaccinate their children”, while 3474 (72.3%) agreed that “Hospitals adequately inform parents of vaccination days/campaigns” and 3617 (75.3%) agreed that “Workshops are needed to raise awareness of fathers concerning the importance of vaccination.” When asked how to improve parents’ motivation to vaccinate, 1847 (38.5%) participants suggested that community-level educational sessions on immunization be held. Other suggestions included “nothing” (n = 736, 15.3%), “improved service quality in health centers (n = 510, 10.6%), “better vaccine coverages” (n = 433, 9.0%), and “vaccination visits in homes” (n = 403, 8.4%).

### Immunization barriers

The 3344 participants with at least one undervaccinated child offered various explanations for their children’s incomplete schedules. These included “the parent not paying attention to the dates on the vaccination card” (n = 1595, 47.7%), “lack of vaccines in health centers” (n = 643, 19.2%), “parental negligence” (n = 365, 10.9%), “expense of vaccines” (n = 360, 10.8%), “lack of time” (n = 247, 7.4%), “the child’s illness following a previous vaccination” (n = 168, 7.5%), and “the child’s lack of affiliation with the health system (n = 90, 2.7%). Using the multivariate factorial techniques, six groups of participants who identified different types of immunization barriers were determined. Characteristics for each group are presented below, with values given in reference to percentages from the overall sample.

#### Group 1: caregivers who identified barriers related to the parent/guardian (24.4%, n = 1172)

Participants in this group primarily identified problems associated with themselves, such as lack of time (68.6% vs. 29.7%) and long lines in health centers (62.1% vs. 42.0%), as potential immunization barriers (Table 3). Indeed, 17.8% of group one caregivers attributed their child’s incomplete immunization schedule to parental negligence (overall 12.6%), while 12.3% attributed it to lack of time (overall 8.4%). More respondents in this group reported living far from health centers (26.1% vs. 19.5%) and fewer said that immunization services were “excellent” or “good” (79.7% vs. 85.5%).

**Table 3:** Immunization barriers, communication preferences, and service quality, according to six groups of caregivers identifying distinct types of immunization barriers: Colombia, May 2010*

**Indicators (agreement with given statement)**	**Total**	***Group 1 (n = 1,172)***	***Group 2 (n = 946)***	***Group 3 (n = 866)***	***Group 4 (n = 642)***	***Group 5 (n = 628)***	***Group 6 (n = 548)***
		**%**	**%**	**%**	**%**	**%**	**%**
***Group 1: Caregivers identifying barriers associated with parents or caregivers***							
One reason that parents do not vaccinate their children are long lines at health centers	42.0	**62.1**	31.8	56.2	23.1	23.7	37.6
One reason that parents do not vaccinate their children is lack of time	29.7	**68.6**	20.1	6.7	30.8	11.6	19.0
My child has an incomplete schedule or has never been vaccinated because I lack the time	8.4	**12.3**	6.5	6.4	8.5	11.4	3.8
My child has an incomplete schedule or has never been vaccinated due to my negligence (or the negligence of the parents)	12.6	**17.8**	9.5	9.4	14.3	11.5	11.8
I have forgotten at least one vaccination appointment	25.5	**31.1**	25.5	21.0	24.1	22.4	26.0
***Group 2: Caregivers identifying barriers associated with vaccinators***							
One reason that parents do not vaccinate their children is the vaccinator’s fear of administering multiple vaccines	2.6	0.0	**13.4**	0.0	0.0	0.0	0.0
One reason that parents do not vaccinate their children is because the vaccinator said the child cannot be immunized if the parent has not brought the child’s vaccination card	5.7	3.3	**11.2**	1.4	10.0	5.1	3.4
One reason that parents do not vaccinate their children is because the vaccinator says the child has the flu and thus cannot be vaccinated	16.5	4.2	**56.9**	5.7	6.0	7.3	13.4
One reason that parents do not vaccinate their children is because the vaccinator says the child has a fever and thus cannot be vaccinated	13.4	2.1	**55.2**	2.8	2.8	3.6	6.0
During my last visit to a health center, the physician reviewed my child’s vaccination card	79.9	82.4	**80.4**	81.1	71.1	79.6	82.7
***Group 3: Caregivers identifying barriers associated with the hospital or health center***							
One reason that parents cannot vaccinate their children is the refusal of health workers to open a vaccine vial without a sufficient number of children	16.2	5.4	11.5	**53.2**	8.6	9.1	5.5
One reason that parents cannot vaccinate their children is because there are no vaccines available when they take their children to be immunized	28.1	22.3	24.6	**65.4**	11.1	22.0	14.6
One reason that parents do not vaccinate their children is because healthcare professionals are unfriendly	13.3	5.0	8.8	**38.7**	7.4	9.2	10.5
During my last to a health center, I was unable to vaccinate my child because health workers were unwilling to open a vaccine vial for my child due to the lack of a sufficient number of children	6.9	5.9	5.7	**13.1**	3.4	6.7	5.2
My child has an incomplete schedule or has never been vaccinated because when I took him to be immunized, no vaccines were available*	18.6	22.4	15.2	**20.4**	11.5	24.2	15.5
***Group 4: Caregivers identifying barriers associated with the health care system***							
One reason that parents do not vaccinate their children is the lack of a vaccination card	14.6	6.4	7.5	3.2	**65.5**	9.5	8.4
One reason that parents cannot or do not vaccinate their children is the lack of association with the healthcare system (unaffiliated status)	13.8	3.1	7.1	4.4	**66.1**	7.4	8.8
One reason that parents cannot or do not vaccinate their children is because the vaccination center is far away	33.4	44.1	15.9	32.9	**49.7**	26.2	30.4
One reason that parents do not vaccinate their children is that they have to pay for the vaccines	14.4	7.3	12.3	19.4	**24.9**	11.0	17.2
During my last attempt to vaccinate my child, I had to travel far to a health center	19.5	26.1	19.1	14.6	**18.0**	17.7	17.2
Not everyone has access to vaccination services✚	7.7	6.3	7.2	7.6	**9.8**	7.3	9.4
***Group 5: Caregivers identifying barriers associated with adverse events***							
One reason that parents do not vaccinate their children is because the children become sick following vaccination	22.5	28.9	10.5	7.8	21.9	**53.5**	17.9
One reason that parents do not vaccinate their children is because the husband does not like the children to become sick following vaccination	9.1	3.7	6.6	5.0	6.1	**35.0**	5.5
One reason that parents do not vaccinate their children is because they fear giving the child multiple vaccines at once	9.7	1.4	6.4	2.4	3.0	**47.8**	8.9
My child has an incomplete vaccination schedule or has never been vaccinated because she became sick the last time she was vaccinated	6.2	4.1	7.3	7.5	7.4	**5.2**	6.5
***Group 6: Caregivers identifying barriers associated with religious and cultural beliefs***							
One reason that parents do not vaccinate their children is because the family does not agree with vaccination	5.8	1.4	7.5	1.9	7.5	8.9	**12.8**
One reason that parents do not vaccinate their children is because people living in rural areas do not need vaccines	5.6	1.2	2.5	1.1	0.7	2.2	**37.2**
One reason that parents do not vaccinate their children is because they have religious and/or cultural beliefs that impede vaccination	5.9	1.4	2.3	0.5	3.2	2.5	**37.4**
My parents were never vaccinated and they never became sick✚	19.0	19.4	17.9	18.1	19.9	18.6	**20.4**
People in rural areas do not vaccines✚	13.4	11.4	12.6	12.8	18.3	13.1	**14.1**
***Communication indicators***							
Television is the one of the best ways to raise awareness about vaccination	51.1	**58.0**	49.3	46.9	48.9	50.7	49.2
Talks/workshops in hospitals are one of the best ways to raise awareness about vaccination	37.3	37.9	37.2	30.7	**48.9**	33.0	38.2
Fliers are one of the best ways to raise awareness about vaccination	25.4	27.1	25.1	23.0	21.3	**32.4**	23.0
The public’s knowledge of vaccination can be improved with more information and by training the community	23.8	18.8	26.5	25.6	24.1	23.8	**26.9**
The public’s knowledge of vaccination can be improved by holding talks/workshops in healthcare establishments	22.4	**26.2**	23.6	21.2	23.2	20.4	15.0
The public’s knowledge of vaccination can be improved by raising awareness of the importance of vaccines	17.8	16.8	15.7	15.1	**25.1**	19.6	16.8
***Service indicators***							
During my last visit to a health center, immunization services were “excellent” or “good”	85.5	**79.7**	88.3	83.7	89.5	90.1	86.1
Healthcare workers tell me my child’s next vaccination appointment°°	91.2	**87.3**	92.3	91.3	93.4	92.9	93.3
Healthcare workers tell me which vaccines/boosters my child has received°°	80.1	78.6	82.9	75.0	82.9	80.3	83.3
I need an appointment to vaccinate my child✚	40.5	32.4	32.5	43.1	**57.3**	41.0	**47.4**
All patients are treated equally in health centers✚	73.2	71.1	**79.1**	70.0	73.8	72.7	72.2
Children receive the same service regardless of where they are from✚	76.0	74.5	78.7	76.2	**72.4**	77.5	77.1

*As described in the methodology, the final step of our factor analysis involved corrected for the oversampling of some regions using sampling weights. Percentages provided in this table reflect these adjustments. Bold values for each group represent significant deviations from rates in the overall sample presented in the second column.

✚For the purposes of this table, totals include respondents who indicated they agreed with statement given. Participants who “neither agreed nor disagreed,” “disagreed,” or chose not to respond were excluded.

°°For the purposes of this table, totals include respondents who indicated that event occurred “always” or “almost always.” Respondents who indicated event occurred “sometimes”, “rarely,” or “never” were excluded.

Group one members resided in Atlántico (33.5% vs. 23.1%) and Santanderes (13.9% vs. 7.7%) and were subsidized users of the healthcare system (77.8% vs. 72.6%). Regarding means of communication, group one members preferred television (58.0% vs. 51.1%) and believed that talks/workshops in health centers would improve the public’s knowledge of vaccination (26.2% vs. 22.4%).

#### Group 2: caregivers who identified barriers related to the vaccinator (19.7%, n = 946)

Nearly twenty percent of participants associated immunization barriers primarily with the vaccinator. These included the vaccinator’s unwillingness to immunize the child due to a cold (56.9% vs. 16.5%) or fever (55.2% vs. 13.4%), fear of administering multiple vaccines at once (13.4% vs. 2.6%), and refusal to immunize a child who lacked a vaccination card (11.2% vs. 5.7%).

Group two members lived predominantly in Bogotá (20.6% vs. 15.6%) and Pacífica (19.1% vs. 16.3%). Forty-six percent were Afro-Colombian (overall 35.0%).

#### Group 3: caregivers who identified barriers related to health centers (18.0%, n = 866)

Group three members cited vaccine stockouts (65.4% vs. 28.1%), the unfriendliness of health workers (38.7% vs. 13.3%), and the vaccinator’s refusal to open a single vial of vaccine for a child (53.2% vs. 16.2%) as immunization barriers. Consistent with these findings, more group three members attributed their children’s incomplete schedules to vaccine stockouts (20.4% vs. 18.6%) and more reported that they had been denied service because the vaccinator would not open a vaccine vial without multiple children present (13.1% vs. 6.9%)

Group three members disproportionately resided in Tolima-Huila-Caquetá (16.6% vs. 6.8%). To improve awareness of immunization, members of this group indicated that loudspeakers were a valuable means of communication (8.0% vs. 3.2%) and that improved service in health centers would increase the population’s motivation to vaccinate (16.4% vs. 10.4%).

#### Group 4: caregivers who identified barriers related to the health system (13.4%, n = 642)

Group four participants cited immunization barriers related to a lack of affiliation with the health system (66.1% vs. 13.8%), distance from the vaccination site (49.7% vs. 33.4%), lack of a vaccination card (65.5% vs. 14.6%), and the cost of vaccines (24.9% vs. 14.4%). In addition, 57.5% indicated that they could not vaccinate their child without an appointment (overall 40.5%). A greater proportion also did not possess vaccination cards (1.7% vs. 1.3%).

Group four members lived in Pacífica (23.9% vs. 16.3%), Antioquia (20.9% vs. 12.8%), and Eje Cafetero (17.6% vs. 5.0%). Elevated proportions of this group were Afro-Colombian (41.7% vs. 35.0%) and indigenous (10.3% vs. 6.8%). Group four participants preferred educational sessions to be held in hospitals (48.9% vs. 37.3%) and communication activities conducted through vaccination days/campaigns (39.8% vs. 31.4%). Twenty-five percent indicated that public’s knowledge of vaccination could be improved by raising awareness of the importance of vaccines (overall 17.8%).

#### Group 5: Caregivers who identified barriers related to adverse events (13.1%, n = 628)

Members of this group identified barriers related to the child’s sickness following immunization (53.5% vs. 22.5%), the husband’s unwillingness to vaccinate the child due to potential adverse reactions (35.0% vs. 9.1%), and the fear of a child receiving multiple vaccines at once (47.8% vs. 9.7%). More group five members reported that their children experienced mild adverse events following vaccination, such as fever (85.3% vs. 81.2%), persistent crying (43.4% vs. 36.3%), and swelling at the injection site (28.6% vs. 22.4%).

Participants in this group lived in Atlántico (25.2% vs. 23.1%), Bogotá (20.3% vs. 15.6%), and Antioquia (16.2% vs. 12.8%). The group’s participants were more likely to believe that vaccination should be publicized through flyers (32.4% vs. 25.4%) and posters (19.5% vs. 15.7%).

#### Group 6: caregivers who identified barriers related to society and culture (11.4%, n = 548)

Participants in this group said that “religious and cultural beliefs might impede vaccination” (37.4% vs. 5.9%) and that “one reason that people do not vaccinate their children is the belief that people in rural areas do not need vaccines” (37.2% vs. 5.6%).

Factors correlated with societal and cultural barriers to immunization include being aged 14–18 years (9.7% vs. 6.7%) and possessing less than a primary education (20.9% vs. 16.8%). Group six participants lived predominantly in Atlántico (19.3%), Bogotá (19.2%), and Antioquia (14.1%) in proportions roughly equal to those in the overall study population. More group six members suggested that better training activities would improve the public’s knowledge of vaccination (26.9% vs. 23.8%).

Groups one, five, and six represented a 48.9% of the users interviewed and were used to inform the design of Colombia’s new immunization communication strategies.

## Discussion

This study was instrumental in better understanding parental practices, attitudes and knowledge, perceived service quality, and preferences for communication strategies in Colombia. The study also helped characterize barriers to immunization, showing that nearly half of the parents and guardians in this study were not adequately demanding vaccination services. Overall, 24.4% of participants primarily associated reasons related to themselves (group one) as immunization barriers, while 24.5% cited adverse events and religious/cultural beliefs (groups five and six). These findings reinforce the notion that re-designed and improved communication strategies on immunization were needed in Colombia to generate increased demand for immunization. Additionally, associations among the types of barriers were observed, including associations between service quality and parental factors, such as forgotten appointments. In this respect, our findings are consistent with a recent assessment of 202 studies on the epidemiology of unvaccinated and undervaccinated children in low- and middle-income countries that found factors associated with undervaccination to be complex and interrelated [13].

While this study is the most comprehensive investigation on undervaccination ever conducted in Colombia, conclusions on coverage rates or the overall quality of the EPI cannot be drawn, since 70% of participants of the study were required to have undervaccinated children. Additionally, attributing non-vaccination or undervaccination to a single cause risks oversimplification [13,14]. For this reason, multivariate analytical techniques were used to categorize participants according to groups of related reasons. A participant may belong to one group, while being influenced by factors in other groups. However, we recognize that the potential overlap among groups risks confusing reasons for under-vaccination. This is particularly true in situations in which a participant may have chosen to attribute a child’s undervaccination to the healthcare system rather than to his own actions. For example, a parent who did not obtain a vaccination card for her child may have attributed her child’s vaccination status solely to a health center’s refusal to vaccinate her child due to lack of a vaccination card. A final set of limitations relates to participant responses. Recall and reporting biases may have affected responses to questions on service quality and attitudes favoring vaccination. In addition, since the multivariable analysis was based on participant beliefs concerning the reasons that “people” do not vaccinate their children, these responses do not necessarily reflect participants’ own reasons for not vaccinating their children.

Despite these limitations, this study provides valuable insights on declining immunization coverage rates in Colombia. Most significantly, caregivers may now be less motivated to vaccinate their children. Indeed, while 48.9% of participants identified immunization barriers related to the demand of vaccines as the primary obstacles to immunization, these factors may play a larger role among caregivers with undervaccinated children. Of the 3344 caregivers studied who had at least one undervaccinated child, 2177 (65.1%) attributed their child’s incomplete schedule to parental negligence, lack of time, or not paying attention to the dates on the vaccination card—all problems related to the demand for rather than the supply of vaccines. Ironically, the success of Colombia’s immunization program in eliminating or significantly reducing the incidence of vaccine-preventable diseases may have contributed to the public’s perception that immunization is now less important. This phenomenon, in which immunization programs become victims of their own success, has been observed in other countries [9].

To increase user motivation, new communication strategies should appeal to the emotions of caregivers, emphasizing that vaccine-preventable diseases still pose serious threats to their children’s wellbeing, while addressing common misperceptions about immunization. To this end, four basic messages for caregivers have been identified. First, children are not fully vaccinated unless they have received all doses of all required vaccines. Second, parents must bring vaccination cards to health centers regardless of the purpose of the visit so that health workers may review the card. Third, vaccination is a shared responsibility between parents and health workers—and parents should be *active* participants in the process. If more parents demand that health workers review the vaccination card, fewer children will leave health centers with incomplete schedules. Lastly, in light of the participants who identified cost as a barrier, the EPI should remind users that all vaccines in the national schedule are free for citizens. While the number of parents attributing their children’s incomplete schedules to vaccine cost was likely elevated because the study was conducted at a time in which PCV was not yet included in the national schedule, reminders emphasizing vaccination as a free public good may increase the population’s motivation to vaccinate.

Another important contribution from this study is the ability to better tailor the EPI communication strategies to the wide differences among immunization services users (audience segmentation). For example, messages can remind those individuals who identify lack of affiliation with the health system as a barrier that neither affiliation, nor the possession of a vaccination card, nor registration in Colombia’s civil registry are requirements for vaccination. Alternatively, the EPI may design strategies to prioritize vulnerable regions identified in this and previous studies [15]. These regions might include Antiguos Territorios Nacionales, Pacífica, Santanderes, and Tolima-Huila, where some service-quality indicators were found to be more than 20 points below national averages.

Attention should also be paid to minority populations. Elevated proportions of Afro-Colombians and indigenous citizens identified barriers related to the vaccinator and health system, and only 72.1% participants believed that people in health centers were treated equally regardless of their affiliation with the healthcare system. These findings are consistent with previous studies in Colombia [5,6]. Another potential target group is fathers. While mothers generally take children to be vaccinated, fathers often influence the decision to vaccinate. Participants in all six groups indicated that workshops should be held to educate fathers on the importance of vaccination.

In addition to the need for tailored interventions, several interventions applicable to all regions and respondent groups were identified. Foremost among these is the potential use of educational sessions to reinforce the four aforementioned messages; 38.5% of participants indicated that such sessions were the best means to raise awareness of vaccination. Ideally, healthcare or public health professionals would lead these sessions in collaboration with community leaders [9]. To complement these efforts, the EPI should seek to improve communication efforts in health centers. Though evidence on the impact of face-to-face interventions for informing caregivers about childhood vaccination is mixed, some studies have suggested that successful interactions between parents and healthcare professionals encourage caregivers to return for immunizations in the future [16,17]. Communication interventions in Colombia should thus work to improve health workers’ ability to establish a rapport with parents, understand and address their concerns, and comply with basic standards of care. It is recommended that the EPI conduct training and supervision activities to teach vaccinators to be better advocates for immunization and prepare them to administer vaccines properly and according to national policy (e.g., opening a vaccine vial for a single child is acceptable). Checklists may help to ensure that health workers provide caregivers with information on the vaccines administered, what to do if a reaction occurs, and when to return to receive the next vaccination dose [18,19].

### Lessons learned

The EPI benefited from outsourcing this study to a professional polling company. The company provided an independent perspective on the country’s challenges and expertise in conducting national health surveys. The qualitative phase of this study was critical. By convening focus groups of parents of undervaccinated children, the EPI and polling company refined research questions, became aware of important local circumstances, and ensured that the language and content of the surveying tools were appropriate. Results of the qualitative phase complemented the quantitative study, providing health officials with useful descriptive data.

## Conclusion

The EPI of Colombia conducted a large study on parental practices on immunization, attitudes and knowledge, perceived service quality, and barriers to immunization. One component of successful immunization programs is the use of communication strategies to address immunization barriers related to parental circumstances (e.g., lack of time), concerns about adverse events, and religious and cultural beliefs. In this study, almost half of users identified such inhibitors as the primary barriers to immunization. Tailored communication strategies informed by this study are being implemented by Colombia’s EPI. Similar studies in the future can help assess the usefulness of these communication strategies in increasing demand for immunization services.

### Endnotes

^a^At the time of the study, the MSPS (Ministerio de Salud y Protección Social) was named the MPS (Ministerio de Protección Social). The current name, MSPS, is used throughout this article.

^b^Administrative coverage is obtained by dividing the number of doses administered to the target population by the estimated target population.

## Abbreviations

EPI: Expanded program on immunization; MSPS in Spanish: Ministry of health and social protection; SGSSS in Spanish: General health and social security system of colombia; BCG: Bacillus calmette-guerin; DPT: Diphtheria, pertussis, and tetanus vaccine; OPV3: Third dose of oral polio vaccine; MMR: Measles, mumps, and rubella vaccine; SPSS: Statistical package for the social sciences software; PCV: Pneumococcal conjugate vaccine.

## Competing interests

The MSPS identified the problem of undervaccination and potentially inadequate communication strategies on vaccination, which are the focus of this study. However, national officials decided to contract a surveying company to minimize the risk of a conflict of interest. The surveying company collected the data and performed an initial independent analysis of the information. Subsequently, officials from the EPI and the Pan American Health Organization reviewed the data and generated the present article. While this analysis is considered objective, we wish to recognize that the Colombian government did pay a surveying company for this analysis and therefore a potential conflict of interest may exist.

## Authors’ contributions

MVG designed the study and helped to develop the surveying tools. DG, MVG, SPT, PB, and MCP participated in the data analysis. SPT drafted the manuscript with contributions from DG, MV, PB, MCP, and MCD-H. SPT prepared Tables 1, 2 and 3 and performed statistical analyses with assistance from MCD-H. All authors read and approved the manuscript.

## Authors’ information

DAG is the manager of Colombia’s EPI. A Colombian physician, MVG served as the manager of Colombia’s immunization program for five years. In 2011, she joined PAHO as a regional immunization advisor. SPT has worked for PAHO since 2008, mostly recently as a consultant on immunization. He is now a second-year student at Vanderbilt School of Medicine. MCP is a Brazilian physician who serves as PAHO’s focus point on immunization in Colombia. PBA is a Peruvian national with a master’s degree in public health. She has 11 years of experience in public health and works as a technical officer with PAHO’s Immunization Project. MCD-H is a Chilean physician with a master’s degree in epidemiology. Since 2004, she has served as a regional immunization advisor for PAHO, overseeing immunization data quality for the organization.

## Pre-publication history

The pre-publication history for this paper can be accessed here:

http://www.biomedcentral.com/1471-2458/14/669/prepub

## Supplementary Material

Additional file 1 Regions defined for the Immunization barriers study, Colombia, 2010. Click here for file
