# Supplementary material for: Understanding the main barriers to immunization in Colombia to better tailor communication strategies
Source: BMC Public Health. 2014 Jun 30;14:669. doi: 10.1186/1471-2458-14-669 (PMC4089932; doi:10.1186/1471-2458-14-669)
Supplement: Additional file 1 — Regions defined for the Immunization barriers study, Colombia, 2010. [file 1471-2458-14-669-S1.docx]

**Regions defined for the Immunization barriers study, Colombia, 2010**

| **Region** | **Sub-region** | **Departments included** | **Geography** | **Population (2)**  **# (%)** | **Economical activities** |
| --- | --- | --- | --- | --- | --- |
| [Amazonia](https://es.wikipedia.org/wiki/Regi%C3%B3n_Amaz%C3%B3nica_(Colombia)) (Antiguos territorios nacionales) |  | Amazonas, Guainía, Guaviare Putumayo, Vaupés y Vichada | Part of the flat Amazon Basin and the Amazon Rainforest of Colombia | 644,949 (1.4%) | Subsistence farming, logging and hunting, and non-industrial fishing |
| [Andina](https://es.wikipedia.org/wiki/Regi%C3%B3n_Andina_(Colombia)) | Antioquia | Antioquia | Located in the northernmost part of the Eastern and Central Colombian Andean ranges and between the northern part of the Cauca River and the middle part of the Magdalena River | 6,066,003 (13.3%) | Industrial, financial and services |
|  | Eje cafetero | Caldas, Risaralda, Quindío |  | 2,453,121 (5.4%) | Agriculture, mining and cattle raising |
|  | Boyacá-Cundinamarca | Boyacá, Cundinamarca | Located in the high and flat lands of the eastern Andes | 3,774,688 (8.2%) | Dairy farming and industrial crops of potatoes, corn, fruits and flowers. |
|  | Bogotá | Bogotá |  | 7,363,782 (16.2%) | The most important center for industrial and, financial activities, and services. |
|  | Tolima –Huila-Caquetá. | Tolima, Huila, Caquetá. | Located between the central and eastern mountain ranges, separated by the Magdalena River | 2,918,577 (6.4%) | Agriculture, textile and industrial, and exploitation of energy reserves (oil) |
|  | Santanderes | Santander del Norte, Santander | Comprises the northern section of the Eastern Cordillera and borders the Venezuelan territory | 3,308,344 (7.3%) | Agricultural and livestock sector, services, especially in the area of ​​transportation and communications.  Exploitation of energy reserves (oil) |
| [Caribe](https://es.wikipedia.org/wiki/Regi%C3%B3n_Caribe_(Colombia)) (Atlántico) |  |  | Consists of a plain called continental Caribbean or Atlantic Coast and the Colombian waters and island territories in the Caribbean sea | 11,067,051 (24.3%)* | Port, agriculture, livestock, tourism, industrial, commercial and financial sector. Exploitation of minerals such as coal, iron nickel, natural gas and salt |
| [Orinoquía](https://es.wikipedia.org/wiki/Regi%C3%B3n_de_la_Orinoqu%C3%ADa_(Colombia)) (Meta-Arauca-Casanare) |  | Meta-Arauca-Casanare | Plain limited by the Orinoco River basin, located in the eastern; it borders Venezuela. | 1,414,083 (3.1%) | Livestock and oil extraction |
| [Pacífica](https://es.wikipedia.org/wiki/Regi%C3%B3n_del_Pac%C3%ADfico_(Colombia)) |  | Chocó, Valle y Nariño | Located on the west by the Pacific Ocean | 6,498,986 (14.3%) | Industrial fishing, logging, mining industrial gold and platinum, livestock and agriculture |

* Includes the archipelago of San Andres and Providencia, which was not included in the study.

Table done by the authors using data from:

1) <http://geoportal.igac.gov.co/mapas_de_colombia/IGAC/Tematicos2012/RegionesGeograficas.pdf>

2) <https://www.dane.gov.co/index.php/poblacion-y-demografia/proyecciones-de-poblacion> to June 30, 2010
